# The Dynamics of Heart Rate Asymmetry and Situational Sleepiness from Evening to Night: The Role of Daytime Sleepiness

**DOI:** 10.3390/biology13100794

**Published:** 2024-10-03

**Authors:** Valeriia Demareva

**Affiliations:** Faculty of Social Sciences, Lobachevsky State University of Nizhny Novgorod, 603950 Nizhny Novgorod, Russia; valeriia.demareva@fsn.unn.ru; Tel.: +7-904-66-49-13

**Keywords:** heart rate asymmetry, daytime sleepiness, situational sleepiness, biological evening, biological night

## Abstract

**Simple Summary:**

This study examined how both daytime and situational sleepiness are associated with heart rate asymmetry (HRA) from evening to night. Fifty participants were divided into two groups based on their levels of daytime sleepiness: ‘Lower Normal’ and ‘Higher Normal’. HRA and situational sleepiness were assessed at 8 P.M., 9 P.M., and 10 P.M. The results showed that individuals with higher daytime sleepiness had lower HRA by 10 P.M., indicating reduced parasympathetic activity and impaired autonomic regulation. Significant correlations were also found between situational sleepiness and HRA metrics in the ‘Higher Normal’ group, particularly in measures of short-term and long-term heart rate variability. These findings suggest that monitoring HRA could help detect drowsiness and manage fatigue, especially in settings where maintaining alertness is critical, such as driving or shift work.

**Abstract:**

The relationship between daytime sleepiness and heart rate asymmetry (HRA) during the transition from evening to night is crucial for understanding autonomic regulation and its implications for alertness. This study aims to investigate how daytime sleepiness influences HRA dynamics from evening to night and how situational sleepiness correlates with HRA metrics. HRA metrics were calculated at 8 P.M., 9 P.M., and 10 P.M. in 50 participants, categorized into ‘Lower Normal’ and ‘Higher Normal’ daytime sleepiness groups based on Epworth Sleepiness Scale (ESS) scores. Situational sleepiness was assessed using the Karolinska Sleepiness Scale (KSS) and Stanford Sleepiness Scale (SSS). The results demonstrated that individuals with ‘Higher Normal’ daytime sleepiness exhibited lower HRA metrics at 10 P.M. compared to those with ‘Lower Normal’ daytime sleepiness, supporting the suggestion that higher daytime sleepiness correlates with reduced parasympathetic activity and diminished autonomic responsiveness. Significant negative correlations between situational sleepiness and HRA metrics were observed in the ‘Higher Normal’ group, particularly with the SSS. Therefore, increased daytime sleepiness affects HRA dynamics by decreasing parasympathetic activity and altering autonomic regulation at the beginning of the biological night (10 P.M.). These findings suggest potential applications for enhancing drowsiness detection and managing fatigue in safety-critical environments.

## 1. Introduction

Sleepiness is a pervasive phenomenon that significantly affects individuals’ daily lives and performance. It is characterized by a strong desire to sleep and can impair cognitive and motor functions, leading to reduced productivity and an increased risk of accidents, particularly in workers [1]. Daytime sleepiness, in particular, has substantial implications for public safety, especially in contexts such as driving, where it can lead to severe consequences due to impaired alertness and reaction times [2,3]. High levels of daytime sleepiness are often indicative of underlying sleep disorders, inadequate sleep, or poor sleep quality. This condition not only affects daytime functioning but also has a cascading effect on physiological processes throughout the evening and night [4].

Situational sleepiness is a dynamic state, influenced by several factors including circadian rhythms [5], sleep deprivation [6], and other factors [7,8]. It varies throughout the day and night, impacting both cognitive performance [9] and physiological homeostasis [10]. Therefore, it is essential to identify and predict sleepiness to mitigate its adverse effects, especially considering its dynamic nature.

Detecting and predicting sleepiness can be approached through self-reports and biomarker analysis. To assess general daytime sleepiness, the Epworth Sleepiness Scale (ESS) [11] is often used. While subjective self-reports such as the Karolinska Sleepiness Scale (KSS) [12] or Stanford Sleepiness Scale (SSS) [13] can be used to evaluate an individual’s situational level of sleepiness, they may have different sensitivities to certain factors (e.g., the presence of a sleep disorder) [14]. The subjective nature of these tests introduces variability, as they rely on personal interpretation and self-assessment, which can be influenced by mood, environment, and other external conditions. At the same time, objective biomarkers may offer a more consistent and quantifiable method of assessment. Among potential biomarkers, heart rate variability (HRV) has proven to be a valid indicator of autonomic nervous system function and has been extensively used to study sleep and sleep-related disorders [15,16].

HRV metrics provide insights into the balance between sympathetic and parasympathetic nervous system activity [17]. Among these metrics, heart rate asymmetry (HRA) has gained attention as it reflects the non-linear dynamics of heart rate changes [18]. HRA is a common phenomenon both in children [19] and adults [20] where heart rate accelerations and decelerations contribute unequally to short-term, long-term, and total HRV, despite both influencing the RR intervals (time intervals between R-wave peaks in the electrocardiogram) changes that create HRV [21,22]. It is important to distinguish HRA from respiratory sinus arrhythmia (RSA), as RSA specifically refers to the cyclical variation in heart rate associated with the breathing cycle, reflecting parasympathetic (vagal) activity. In contrast, HRA captures broader asymmetrical patterns in heart rate changes, which are not necessarily tied to respiration and may reflect more complex autonomic interactions. Recent studies have demonstrated the potential of HRA measures in differentiating autonomic responses, highlighting their sensitivity to physiological changes, e.g., after an orthostatic challenge [23,24] or isometric handgrip test [25]. These findings underscore the dynamic nature of autonomic regulation in response to physiological stressors, which may similarly influence situational sleepiness throughout the evening and night.

Research on HRA suggests that the magnitude of heart rate accelerations and decelerations could play a significant role in understanding situational and daytime sleepiness. Studies have shown that variations in the intensity and duration of these accelerations and decelerations can reflect changes in autonomic nervous system activity under different physiological conditions [18,24,26]. For instance, distinct patterns in heart rate variability were observed among individuals with obstructive sleep apnea, indicating that decreases in short acceleration and deceleration runs may correspond to increased fatigue or altered autonomic regulation [27]. Similarly, research has found that reductions in short acceleration runs, combined with increases in long acceleration and deceleration runs, were associated with shifts in physiological states [26]. These changes may reflect alterations in autonomic regulation, potentially linked to variations in situational and daytime sleepiness. However, the exact nature of this relationship remains unclear, as the mechanisms underlying these heart rate patterns and their connection to sleepiness are not yet fully understood.

Our prior findings demonstrated that in people with ‘Higher Normal’ daytime sleepiness, the subjective sleepiness score did not change from the biological evening (8 P.M.) to biological night (10 P.M.). At the same time, the sympatho-vagal index—indicating the balance between sympathetic and parasympathetic activity—decreased, and fragmentation heart rate metrics increased from 8 P.M. to 10 P.M., suggesting that their transition from evening to night mode involves a reduction in body (physical arousal) and cognitive (mental strain) tension [28]. This may reflect a more balanced influence of the sympathetic and parasympathetic systems at the beginning of the biological night, leading to lowering HRA. But this suggestion needs to be verified.

Given the dynamic nature of situational sleepiness and its significant impact on daily functioning and safety, understanding the interplay between daytime and situational sleepiness and their combined effect on HRA needs to be studied. By analyzing HRA metrics, it is possible to better understand the physiological mechanisms underlying sleepiness. Therefore, it has been proposed that heart rate variability, particularly HRA, is a promising candidate for predicting and understanding sleepiness dynamics.

The primary objective of this study is to investigate the dynamics of HRA and situational sleepiness from evening to night and to understand the role of daytime sleepiness in these dynamics. By examining HRA metrics at 60 min intervals, we aimed to identify the temporal patterns of autonomic regulation and situational sleepiness in individuals with different levels of daytime sleepiness. It was hypothesized that individuals with higher daytime sleepiness would show lower HRA at the beginning of the biological night, and that HRA metrics would correlate with situational sleepiness, particularly in people with higher daytime sleepiness. Better understanding of cardiovascular function can help to offer innovative therapeutic targets for hypertensive patients with sleep-related conditions [29].

## 2. Materials and Methods

### 2.1. Study Design

In this experiment, the Subjective Sleepiness Dynamics Dataset (SSDD) was utilized, which was gathered as part of a previous study [14]. At the time of this paper’s preparation, the SSDD contained data from 240 participants. The SSDD focuses on collecting information on sociodemographic characteristics, daytime and situational sleepiness, sleep quality, and heart rate recordings from 8 P.M. to 6 A.M. in individuals over 18 years old. Data collection was conducted over a 15-month period, without any follow-up after the main collection phase. For this study, data from 8 P.M. to 10 P.M. over a three-month period, from September to November 2023, were selected.

This study is a single-center, observational, prospective study aimed at investigating the relationship between HRA metrics and situational sleepiness across different levels of daytime sleepiness.

### 2.2. Study Setting

This study was carried out in a community setting, recruiting participants from Nizhny Novgorod Region through local and regional news portals. The participants completed this study at home, with specific instructions to avoid physical exercise and adhere to their usual household activities during the experimental period.

### 2.3. Participants

Participants were selected from the SSDD based on the following inclusion criteria: healthy adults aged between 18 and 60 years (per WHO classification [30]), with no history of chronic diseases, and no use of prescribed medications. Only participants with an average level of daily physical activity (neither sedentary nor professional athletes) were included. 

Physical activity level was assessed based on the participants’ reported daily routines. For the purpose of this study, participants were considered to have ‘average physical activity’ if their routines included light to moderate activities, such as walking or performing household chores but excluded activities like professional athletic training. For instance, using stairs in a building with a lift was not considered an additional physical activity, unless participants deliberately avoided the lift. 

The exclusion criteria included recordings where the time of cyclic tests (KSS and SSS) differed by more than 10 minutes from the specified times (8 P.M., 9 P.M., and 10 P.M.), going to bed outside the 10:30 P.M. to 12 A.M. window, and poor-quality heart rate data. Additionally, participants with severe levels of daytime sleepiness (ESS scores more than 10 points) were excluded. Pregnancy was also an exclusion criterion.

Thus, 50 participants with ‘Lower Normal’ daytime sleepiness (ESS score 0–5, *n* = 27) and ‘Higher Normal’ daytime sleepiness (ESS score 6–10, *n* = 23) were selected for the analysis. The age and gender distribution of the sample is presented in Table 1.

### 2.4. Sampling and Recruitment

The participants were recruited through local news portals and completed an initial screening via a Google Form, where they provided basic demographic information (age and gender) and a phone number. The experimenters contacted the participants and invited them to the laboratory, where they signed an informed consent form. They were informed that they could withdraw from the experiment at any time without providing a reason. In the laboratory, they were shown how the equipment works, provided with the necessary devices, and given instructions on how to use them.

The sampling process targeted a total of 50 participants who met the inclusion criteria mentioned in Section 2.3.

### 2.5. Outcomes and Measures

The primary outcome of this study was the change in HRA metrics, including SD1, SD2, C1d, C1a, C2d, C2a, GI, SI, PI, and AI across three time points (8 P.M., 9 P.M., and 10 P.M.). 

SD1 measures short-term variability by calculating the standard deviation of RR intervals perpendicular to the line of identity, while SD2 represents long-term variability by calculating the standard deviation along the line of identity. C1d and C1a reflect the contributions of heart rate decelerations and accelerations to short-term variability, respectively, whereas C2d and C2a reflect the contributions of decelerations and accelerations to long-term variability. Guzik’s Index (GI) is the ratio of the distance of points above the line of identity to the total distance of all points, except those on the line. The Slope Index (SI) is the ratio of the phase angle of points above the line of identity to the phase angle of all points except those on the line. Porta’s Index (PI) is the ratio of points below the line of identity to the total number of points, excluding those on the line. Lastly, the Area Index (AI) is the ratio of the cumulative area of points above the line of identity to the cumulative area of all points except those on the line. The description of these metrics is additionally provided in Table A1 (Appendix A).

Secondary outcomes included the participants’ daytime (ESS) and situational sleepiness scores (KSS and SSS) and their correlation with HRA metrics. The data were analyzed at three time points (8 P.M., 9 P.M., and 10 P.M.), with HRA and situational sleepiness scores gathered through cyclic tests.

Sociodemographic information and sleepiness data were collected using the web application UnnCyberpsy, developed with the programming language Hypertext PreProcessor (PHP, version 8.1) based on the microframework CodeIgniter, version 4 (British Columbia Institute of Technology, Burnaby, BC, Canada). CodeIgniter is an open-source framework for web application development. Data storage was managed with ‘MariaDB’, an open-source relational database management system.

Heart rate intervals were recorded using the Polar H10 sensor and the Pro Strap belt (Polar Electro Oy, Kempele, Finland). The Polar H10 sensor’s validity has been demonstrated in several studies (e.g., [31,32]). The data were transmitted to the Polar Sensor Logger App v. 0.25 (Jukka Happonen, Helsinki, Finland) on a Samsung A23 smartphone (Samsung Electronics Co., Ltd., Suwon, Republic of Korea) via Bluetooth. The data were then transferred to a laptop for further analysis.

### 2.6. Data Collection Procedures

**Figure 1:**
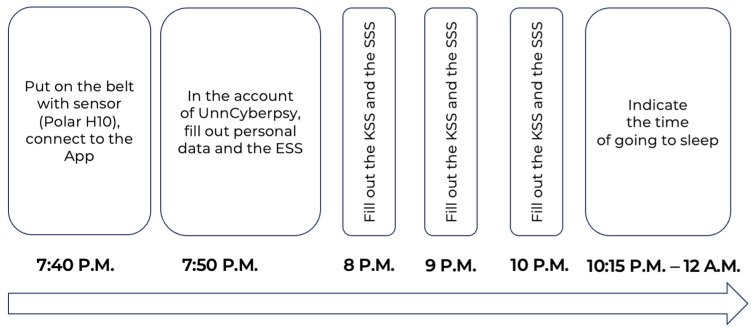
Experimental protocol.

The participants were instructed to connect the Polar H10 sensor to the Polar Sensor Logger App at 7:40 P.M., following which they completed sociodemographic forms and the ESS [11] through the application UnnCyberpsy. From 8 P.M., the participants began completing cyclic tests (KSS [12,33] and SSS [13]) every 30 min until their bedtime and specified the time of going to bed. For the purpose of this study, only the data at 8 P.M., 9 P.M., and 10 P.M. were selected; see Figure 1.

### 2.7. Data Management

All collected data were securely stored in encrypted databases, with the participants assigned anonymized IDs to ensure confidentiality. Incomplete or missing data points were addressed using standard imputation methods, excluding data where necessary based on predefined criteria.

### 2.8. Data Analysis

Data preprocessing was carried out using Jupyter Notebook within the Anaconda 2020.07 (Python 3.8.3, 64-bit) distribution [34]. RR intervals below 400 ms and above 1300 ms, or those differing by more than 70% from the median of the previous five intervals, were removed.

To compute heart rate asymmetry (HRA) metrics, the Neurokit2 Python module [35] was employed. Neurokit2 is an open-source Python toolbox specifically designed for bio-signal processing, including electrocardiogram (ECG) data. It offers advanced features for HRA metric calculation. For this study, the module Neurokit2 was used to calculate key HRA metrics including SD1, SD2, C1d, C1a, C2d, C2a, GI, SI, PI, and AI at each of the three time points (8 P.M., 9 P.M., and 10 P.M.) using measurements obtained over a 10-minute period.

Statistical analysis was performed using the ’Pingouin’ [36], ’Scipy’ [37], and ’Statsmodels’ [38] Python packages. The normality of distributions was assessed using the Shapiro–Wilk test and the equality of variances using Levene’s test. Differences between ESS levels and time points were assessed using two-way repeated-measures ANOVA, with post hoc Sidak correction for multiple comparisons. A linear mixed-effects model was applied to investigate the relationship between age (18–25 y.o., 26–35 y.o., 36–45 y.o., and 45–60 y.o.), sex (male and female), and significant HRA metrics across three time points (8 P.M., 9 P.M., and 10 P.M.). Correlations between subjective sleepiness ratings (KSS, SSS) and HRA metrics were examined using Pearson correlation at each time point. We did not have any missing data points in the present study.

For HRA metrics with statistically significant differences observed in the two-way repeated-measures ANOVA, further modeling using a linear mixed-effects model was conducted to assess the influence of demographic factors (age and sex) on these metrics over time.

All analyses considered a p-value less than 0.05 as statistically significant.

### 2.9. Ethical Considerations

This study received ethical approval from the Ethics Committee of Lobachevsky State University. All the participants provided written informed consent in accordance with the Declaration of Helsinki. 

## 3. Results

**Figure 2:**
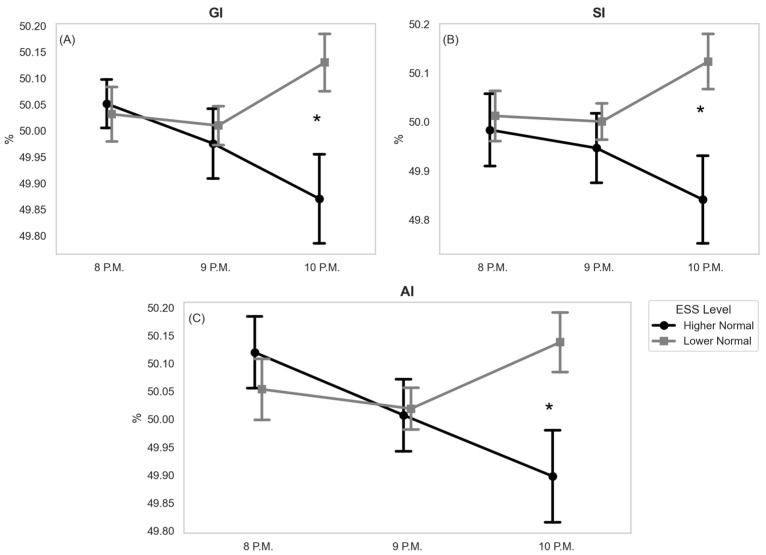
Mean values ± SEM of HRA metrics for ‘Higher Normal’ (*n* = 23) and ‘Lower Normal’ (*n* = 27) ESS levels at 8 P.M., 9 P.M., and 10 P.M.; (**A**) Guzik’s Index (GI), (**B**) Slope Index (SI), (**C**) Area Index (AI). *—*p* < 0.05. Differences in means were analyzed using a Sidak post hoc test.

The analysis did not identify significant effects of ESS levels (F(1, 48) = 2.61, *p* = 0.113, η^2^ = 0.025) or time (F(2, 96) = 0.43, *p* = 0.652, η^2^ = 0.005) on GI. The interaction effect between ESS level and time was significant, F(2, 96) = 4.21, *p* = 0.018, η^2^ = 0.044. There also were no effects on ESS levels (F(1, 48) = 3.47, *p* = 0.069, η^2^ = 0.037) or time (F(2, 96) = 0.10, *p* = 0.90, η^2^ = 0.001) on SI identified. The interaction effect between ESS levels and time was significant, F(2, 96) = 3.42, *p* = 0.037, η^2^ = 0.033. The analysis revealed no significant main effects of ESS levels (F(1, 48) = 1.31, *p* = 0.257, η^2^ = 0.011) or time (F(2, 96) = 0.89, *p* = 0.424, η^2^ = 0.011) on AI. The interaction effect between ESS levels and time was significant, F(2, 96) = 3.99, *p* = 0.022, η^2^ = 0.047, indicating that the effect of time on AI depended on the ESS levels. No other significant main or interaction effects were found. Figure 2 shows the mean values ± SEM (standard error of the mean) of HRA metrics (GI, SI, and AI) for ‘Higher Normal’ and ‘Lower Normal’ ESS levels at 8 P.M., 9 P.M., and 10 P.M.

The results of the linear mixed-effects regression analysis (see Table A2, Table A3 and Table A4) showed that sex and age had minimal impact on HRA metrics (GI, SI, and AI) across the studied time points (8 P.M., 9 P.M., and 10 P.M.). Specifically, the effects of sex were non-significant for both GI (*t* = 1.67) and SI (*t* = 0.92), with only a modest influence observed on AI (*t* = 2.48). Regarding age, none of the age groups showed significant differences in GI or SI, except for a minor effect of the oldest age group (more than 45 years) on both GI (*t* = −2.05) and SI (*t* = −2.24). However, for AI, age did not show any significant effect. The time of measurement itself also had negligible effects, with all time points showing non-significant changes across all three metrics.

**Figure 3:**
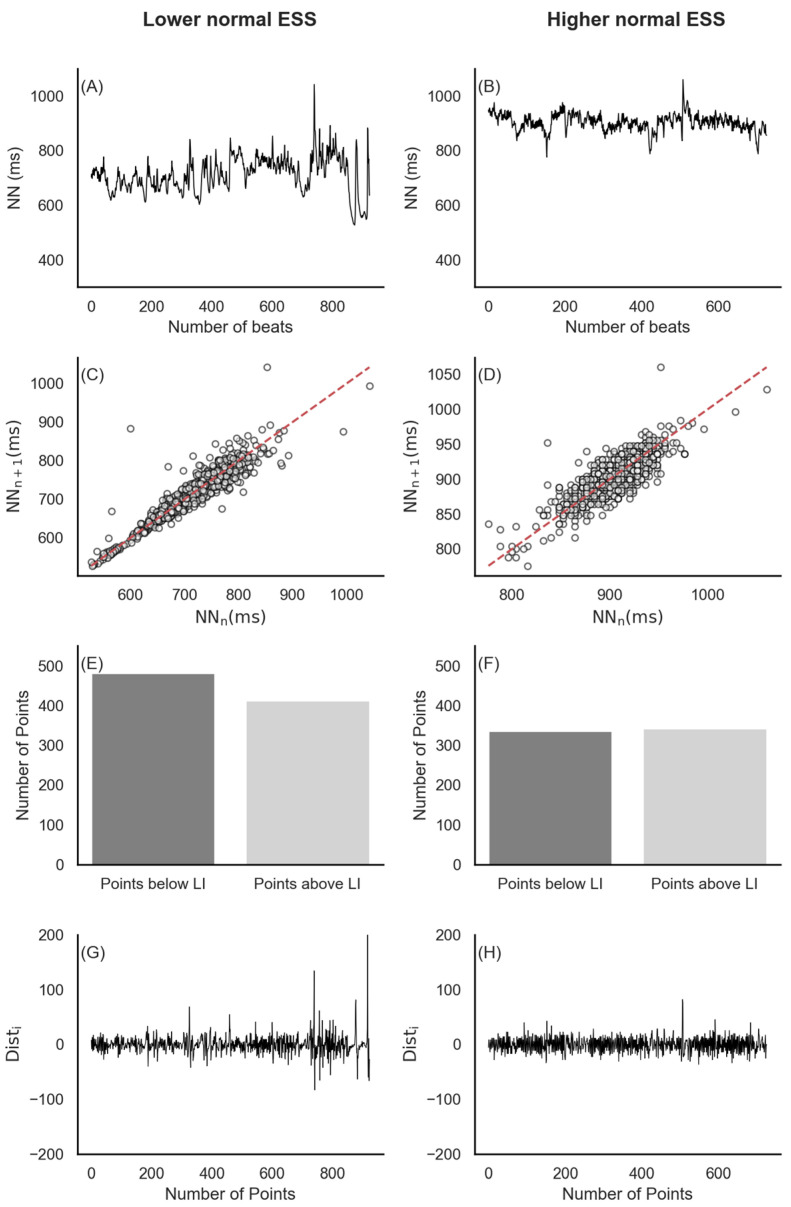
An example of NN interval traces (**A**,**B**), Poincaré Plots (**C**,**D**), distribution of points below and above LI (**E**,**F**), and Dist_i_ (**G**,**H**) at 10 P.M. for participants with ‘Lower Normal’ and ‘Higher Normal’ daytime sleepiness.

Figure 3 illustrates typical NN interval traces for one participant from each group at 10 P.M. (A and B). Subplots C and D display the corresponding Poincaré plots for the NN intervals. The number of points above and below the line of identity (LI) in subplots E and F correspond to the Poincaré plots shown in subplots C and D. Subplots G and H depict the distance (Dist_i_) of each point from LI in the Poincaré subplots.

Table 2 and Table 3 demonstrate the results of correlation analysis between situational sleepiness ratings (the KSS and the SSS) and HRA metrics at each time point (8 P.M., 9 P.M., and 10 P.M.) in ‘Lower Normal’ and ‘Higher Normal’ daytime sleepiness, respectively.

No significant correlations were observed for ‘Lower Normal’ daytime sleepiness group. For ‘Higher Normal’ daytime sleepiness, significant correlations were found only for the SSS: at 8 P.M. with SD1 (r(21) = −0.49, *p* = 0.017) and SD2 (r(21) = −0.50, *p* = 0.015); at 9 P.M. with C2d (r(21) = −0.42, *p* = 0.046) and C2a (r(21) = 0.42, *p* = 0.046).

## 4. Discussion

This study examined differences in heart rate asymmetry (HRA) at 8 P.M., 9 P.M., and 10 P.M. among individuals with varying levels of daytime sleepiness, taking into account their situational sleepiness at these times. The analysis focused on participants with ‘Lower Normal’ and ‘Higher Normal’ levels of daytime sleepiness, as classified by their ESS [11] scores. Situational sleepiness was measured using both the KSS [12,33] and the SSS [13]. Previous research suggests that individuals with higher daytime sleepiness require additional physiological resources to remain awake throughout the day, which may result in decreased autonomic balance between sympathetic and parasympathetic activity at the beginning of the biological night (10 P.M.). Moreover, HRA metrics are known to be sensitive indicators of physiological changes [23,24,25]. Our findings reveal that the transition to the biological night (10 P.M.) affects these two groups differently. At 10 P.M., the participants in the ‘Higher Normal’ group exhibited lower HRA metrics (GI, SI, and AI) compared to those in the ‘Lower Normal’ group, supporting our first hypothesis. Specifically, individuals with higher daytime sleepiness showed reduced HRA at the beginning of the biological night.

GI measures the frequency and magnitude of decelerations in heart rate, reflecting parasympathetic nervous system activity [23,39]. Lower GI in people with ‘Higher Normal’ daytime sleepiness at 10 P.M. implies fewer and less pronounced decelerations, which could indicate diminished parasympathetic regulation or a less responsive autonomic system. Given our previous results, which showed that in people with ‘Higher Normal’ daytime sleepiness, the autonomic balance index decreases from 8 P.M. to 10 P.M. [24], it can be assumed that this decrease is primarily due to a reduction in sympathetic nervous system activity rather than an increase in parasympathetic branch activity. Current findings can be compared to studies examining heart rate variability in individuals with obstructive sleep apnea (OSA). For instance, distinct patterns in heart rate variability were observed among individuals with OSA, where decreases in short acceleration and deceleration runs were associated with increased fatigue or altered autonomic regulation [26]. The current results are also consistent with the finding that severe OSA patients have fewer short deceleration runs [26], as a lower GI indicates fewer and less pronounced decelerations.

The lower AI values in individuals with ’Higher Normal’ daytime sleepiness suggest a reduction in heart rate variability [37]. This decreased variability may be associated with a more stable but less flexible state of autonomic regulation in the evening. AI, therefore, reasonably corresponds to patterns observed in OSA [24,25], reflecting overall changes in heart rate dynamics. These findings suggest that reduced parasympathetic activity and diminished autonomic responsiveness may occur in individuals with higher daytime sleepiness as the evening progresses.

OSA is typically associated with sympathetic excitation due to positive chemoreflex activation [40], which results in an increase in heart rate and reduced parasympathetic activity during apneic episodes. This leads to fewer decelerations and an overall higher autonomic balance index. In contrast, our previous results [24] showed a decrease in the autonomic balance index in individuals with ’Higher Normal’ daytime sleepiness from 8 P.M. to 10 P.M., which we attribute to a reduction in sympathetic nervous system activity rather than parasympathetic activation. This suggests that, unlike in OSA where sympathetic excitation is driven by hypoxia and chemoreflex, the decrease in autonomic balance in our previous study [24] may reflect a natural transition towards relaxation and reduced physiological arousal as the participants prepare for sleep. Therefore, while the lower GI in both OSA and our current study reflects fewer decelerations, the underlying cause differs, with OSA being driven by pathological mechanisms, while our findings suggest a response to normal sleep–wake processes.

The present study also examined the potential influence of gender and age on HRA metrics. The results of the linear mixed-effects regression analysis indicated that gender and age did not significantly affect the key HRA metrics (GI, SI, and AI) across the time points studied. While a modest influence of gender on AI was observed (bigger values for males, which supports the findings reported in [20]), the overall impact was minimal, and no significant differences were found in the other metrics.

When considering the influence of age, it is important to note that autonomic tone, particularly parasympathetic function, tends to decline with aging [41]. In our study, which included individuals between 18 and 60 years old, the modest decrease in GI and SI observed in the oldest age group (over 45 years) is consistent with well-documented age-related reductions in parasympathetic regulation, including a decrease in RSA [42]. Although RSA primarily reflects parasympathetic tone, its diminishing influence with age could lead to less variability in heart rate dynamics overall, which might partially explain the reduction in GI and SI in the older participants.

However, in this study, the lack of a substantial age effect on HRA metrics (GI, SI, AI) across most age groups suggests that the primary drivers of heart rate asymmetry in our sample are related to variations in situational and daytime sleepiness rather than to age-related changes in autonomic function. While a decrease in parasympathetic activity and reduced autonomic responsiveness with age is expected, our results indicate that sleepiness levels, rather than aging itself, are the more significant contributors to the observed HRA dynamics during the transition into the night period.

Furthermore, the influence of RSA on heart rate variability may interact with sleepiness levels, but the evening reduction in HRA observed in individuals with ‘Higher Normal’ daytime sleepiness likely reflects changes in both sympathetic and parasympathetic balance as the body transitions towards rest. Thus, while RSA and age-related changes in parasympathetic tone are important considerations, the data suggest that the dynamics of sleepiness and autonomic regulation in the evening play a more prominent role in shaping HRA patterns in this study population.

Further, the correlations between daytime sleepiness levels and several HRA metrics were examined. Notably, different patterns of correlations were observed for the participants in the ‘Lower Normal’ and ‘Higher Normal’ daytime sleepiness groups. For the participants classified in the ‘Lower Normal’ daytime sleepiness group, no significant correlations were found between situational sleepiness and the heart rate variability indices at 8 P.M., 9 P.M., or 10 P.M. This lack of significant correlation suggests that within this group, HRA measures may not be strongly influenced by variations in situational sleepiness. In contrast, for the participants with ‘Higher Normal’ daytime sleepiness, significant correlations were found between situational sleepiness and specific HRA metrics. These results confirm the second hypothesis of this study about the correlation of HRA metrics with situational sleepiness ratings in individuals with higher daytime sleepiness.

At 8 P.M., significant negative correlations were observed between the SSS and both SD1 and SD2. SD1 and SD2 represent short-term and long-term heart rate variability, respectively, both of which have been linked to physical fatigue [43]. The negative correlations suggest that higher situational sleepiness at 8 P.M. is associated with a decrease in both short-term and long-term heart rate variability. This reduction in variability may indicate a decline in autonomic responsiveness and an increase in physical fatigue.

At 9 P.M., significant negative correlations were found between the SSS and both C2d and C2a. C2d reflects the contribution of heart rate decelerations to long-term HRV, while C2a reflects the contribution of accelerations to long-term HRV. These results suggest that higher situational sleepiness at 9 P.M. is associated with a reduced impact of decelerations and an increased contribution of accelerations to long-term HRV. This shift in heart rate dynamics indicates that increased situational sleepiness may alter autonomic regulation, with a tendency towards more frequent accelerations and fewer decelerations as individuals with ‘Higher Normal’ daytime sleepiness transition into the night period.

It is worth noting that significant correlations between situational sleepiness and HRA metrics were observed only for the SSS. This finding is consistent with previous research suggesting that the SSS may be more sensitive to certain factors compared to the KSS [14].

Therefore, significant correlations were found between situational sleepiness and HRA metrics only in the ‘Higher Normal’ group, with the SSS showing negative correlations with short-term and long-term variability metrics. These findings indicate that higher daytime sleepiness may lead to altered heart rate dynamics and reduced autonomic flexibility during the evening.

## 5. Limitations

This study utilized a sample of 50 participants, which, while sufficient for preliminary analysis, may limit the generalizability of the findings. The specific age and sex distribution (Table 1) reflects a limited group, potentially affecting the broader applicability of the results. Future research with a larger and more diverse sample could enhance the generalizability and robustness of the findings.

The participants were selected based on specific criteria, including bedtime window, age (below 60 years), and absence of chronic diseases. This restricted participant pool may limit the applicability of the results to other age groups or individuals with chronic diseases. Further research is needed to study these groups, as well as adding people with more severe daytime sleepiness levels.

While this study provides valuable insights, it is important to note that social behaviors, lifestyle habits, and eating habits were not fully explored in the methodology. Future research could benefit from considering these factors, as they may further enrich the understanding of the results.

This study focused on recordings between 8 P.M. and 10 P.M. While this timeframe was chosen to control for bedtime variations and to study the transition from the biological evening to biological night, it does not account for potential variations in HRA dynamics throughout the entire night. Including a broader range of time points or extending the measurement period could provide a more comprehensive understanding of how HRA and daytime sleepiness interact over time.

This study represents a pioneering investigation into the relationship between HRA and various levels of daytime and situational sleepiness. As a pilot study, it serves as an initial exploration of how these factors interact, providing foundational insights that can guide future research in this field.

## 6. Conclusions

This study explored the impact of daytime sleepiness on heart rate asymmetry (HRA) metrics and situational sleepiness at 8 P.M., 9 P.M., and 10 P.M. The participants with ‘Higher Normal’ daytime sleepiness exhibited lower HRA metrics—specifically Guzik’s Index (GI), Slope Index (SI), and Area Index (AI)—at 10 P.M. compared to those with ‘Lower Normal’ daytime sleepiness. Significant correlations were found between situational sleepiness and HRA metrics only in the ‘Higher Normal’ daytime sleepiness group, particularly in measures of short-term and long-term heart rate variability. These results may have practical implications for developing tools aimed at detecting drowsiness and enhancing fatigue management, particularly in settings where maintaining alertness is crucial, such as in sport, transportation, and shift work.

## Figures and Tables

**Table 1:** Age and sex distribution of the dataset.

Age	Females (*n*)	Males (*n*)	Total
18–25 y.o.	7	3	10
26–35 y.o.	11	4	15
36–45 y.o.	6	7	13
45–60 y.o.	7	5	12
Total	31	19	50

**Table 2:** The values of Pearson correlation criteria between situational sleepiness ratings (the KSS and the SSS) and HRA metrics at each time point (8 P.M., 9 P.M., and 10 P.M.) in ‘Lower Normal’ daytime sleepiness.

Situational Sleepiness Rating	Time Point	SD1	SD2	C1d	C1a	C2d	C2a	GI	SI	PI	AI
KSS	8 P.M.	−0.02	0.28	0.02	−0.02	−0.03	0.03	−0.08	−0.10	0.08	−0.06
	9 P.M.	−0.10	−0.07	0.06	−0.06	0.00	−0.00	0.21	0.19	−0.01	0.22
	10 P.M.	−0.10	0.27	0.08	−0.08	−0.18	0.18	−0.11	−0.12	0.00	−0.10
SSS	8 P.M.	−0.20	0.02	−0.26	0.26	0.13	−0.13	−0.10	−0.08	−0.11	−0.11
	9 P.M.	−0.02	−0.11	−0.03	0.03	0.15	−0.15	0.17	0.15	−0.05	0.18
	10 P.M.	−0.03	0.21	0.02	−0.02	−0.14	0.14	−0.11	−0.11	−0.04	−0.11

**Table 3:** The values of Pearson correlation criteria between situational sleepiness ratings (the KSS and the SSS) and HRA metrics at each time point (8 P.M., 9 P.M., and 10 P.M.) in ‘Higher Normal’ daytime sleepiness, *—*p* < 0.05.

Situational Sleepiness Rating	Time Point	SD1	SD2	C1d	C1a	C2d	C2a	GI	SI	PI	AI
KSS	8 P.M.	−0.27	−0.30	−0.00	0.00	−0.10	0.10	−0.34	−0.13	−0.04	−0.34
	9 P.M.	−0.08	0.13	0.19	−0.19	−0.32	0.32	−0.09	−0.11	0.12	−0.06
	10 P.M.	−0.11	0.03	0.11	−0.11	−0.09	0.09	−0.08	−0.09	−0.08	−0.07
SSS	8 P.M.	−0.49 *	−0.50 *	−0.22	0.22	0.28	−0.28	−0.02	0.19	−0.14	−0.24
	9 P.M.	−0.15	0.11	0.34	−0.34	−0.42 *	0.42 *	0.02	0.01	0.37	0.04
	10 P.M.	−0.06	−0.21	0.06	−0.06	−0.23	0.23	−0.00	−0.00	−0.06	−0.00

## Data Availability

The data presented in this study are available on request from the corresponding author. The data are not publicly available due to their containing information that could compromise the privacy of the research participants.

## Appendix A

**Table A1:** Descriptions of heart rate asymmetry metrics (taken from [39]).

Metrics	Description
SD1	Standard deviation perpendicular to the line of identity. It is an index of short-term RR interval fluctuations, i.e., beat-to-beat variability. It is equivalent (although on another scale) to RMSSD, and therefore, it is redundant to report correlations with both.
SD2	Standard deviation along the identity line. Index of long-term HRV changes.
C1d	Contributions of heart rate decelerations to short-term HRV
C1a	Contributions of heart rate accelerations to short-term HRV
C2d	Contributions of heart rate decelerations to long-term HRV
C2a	Contributions of heart rate accelerations to long-term HRV
GI	Guzik’s Index, defined as the distance of points above the line of identity (LI) to the LI divided by the distance of all points in a Poincaré plot to the LI except those that are located on the LI
SI	Slope Index, defined as the phase angle of points above the LI divided by the phase angle of all points in a Poincaré plot except those that are located on the LI
PI	Porta’s Index, defined as the number of points below the LI divided by the total number of points in a Poincaré plot except those that are located on the LI
AI	Area Index, defined as the cumulative area of the sectors corresponding to the points that are located above the LI divided by the cumulative area of the sectors corresponding to all points in a Poincaré plot except those that are located on the LI

**Table A2:** The results of the linear mixed-effects regression analysis of GI.

Fixed Effect	Estimate	Std. Error	*t* Value
Intercept	50.02	0.07	735.88
Gender (male)	0.09	0.06	1.67
Age group (26–35 years)	0.04	0.08	0.59
Age group (36–45 years)	0.05	0.08	0.61
Age group (more than 45 years)	−0.16	0.08	−2.05
Time (9 P.M.)	−0.05	0.05	−0.89
Time (10 P.M.)	−0.03	0.05	−0.58

**Table A3:** The results of the linear mixed-effects regression analysis of SI.

Fixed Effect	Estimate	Std. Error	*t* Value
Intercept	50.00	0.08	637.61
Gender (male)	0.06	0.07	0.92
Age group (26–35 years)	0.04	0.09	0.40
Age group (36–45 years)	0.05	0.09	0.49
Age group (more than 45 years)	−0.21	0.09	−2.24
Time (9 P.M.)	−0.02	0.05	−0.43
Time (10 P.M.)	−0.01	0.05	−0.10

**Table A4:** The results of the linear mixed-effects regression analysis of AI.

Fixed Effect	Estimate	Std. Error	*t* Value
Intercept	50.03	0.07	756.25
Gender (male)	0.13	0.05	2.48
Age group (26–35 years)	0.06	0.07	0.78
Age group (36–45 years)	0.05	0.07	0.69
Age group (more than 45 years)	−0.11	0.07	−1.53
Time (9 P.M.)	−0.07	0.06	−1.22
Time (10 P.M.)	−0.06	0.06	−0.98
